# Detection of KPC-216, a Novel KPC-3 Variant, in a Clinical Isolate of *Klebsiella pneumoniae* ST101 Co-Resistant to Ceftazidime-Avibactam and Cefiderocol

**DOI:** 10.3390/antibiotics13060507

**Published:** 2024-05-29

**Authors:** Maria Giufrè, Giulia Errico, Maria Del Grosso, Michela Pagnotta, Bernardetta Palazzotti, Milva Ballardini, Annalisa Pantosti, Marcello Meledandri, Monica Monaco

**Affiliations:** 1 Department of Infectious Diseases, Istituto Superiore di Sanità, 00161 Rome, Italy; 2 San Filippo Neri Hospital, 00135 Rome, Italy

**Keywords:** ceftazidime-avibactam, cefiderocol, cross-resistance, KPC-216, ESKAPE

## Abstract

Background: Carbapenemase-producing *Klebsiella pneumoniae* (CP-KP) represents a global threat to public health, with limited antimicrobial therapeutic options. In this study, we analyzed a ceftazidime/avibactam (CAZ-AVI)-resistant *K. pneumoniae* isolate obtained from a patient previously exposed to CAZ-AVI expressing a novel *K. pneumoniae* carbapenemase (KPC)-3 variant. Methods: Antimicrobial susceptibility testing was performed using reference broth microdilution. Whole-genome sequencing (WGS) was performed using Illumina and Nanopore Technologies. Short- and long-reads were combined with Unicycler. Assemblies were investigated for multilocus sequence typing (MLST), antimicrobial resistance genes, porins, and plasmids. Results: The *K*. *pneumoniae* isolate (KP_RM_1) was resistant to CAZ-AVI, expanded-spectrum cephalosporins, amikacin, ertapenem, and cefiderocol (FDC) but was susceptible to tigecycline, colistin, trimethoprim/sulfamethoxazole, meropenem–vaborbactam, and imipenem–relebactam. WGS revealed that the KP_RM_1 genome is composed of a single chromosome of 5 Mbp and five circular plasmids. Further analysis showed the presence of novel *bla*_KPC-216_ located on a 72 kb plasmid. KPC-216 differs from KPC-3 by a Lysin (K) insertion at position 168 (+K168). Conclusions: We report the identification of a new KPC-3 variant associated with CAZ-AVI resistance. The KPC variants associated with CAZ-AVI resistance should be determined to promptly inform clinicians and start the appropriate antimicrobial therapy.

## 1. Introduction

During the last decade, the worldwide spread of carbapenemase-producing *Klebsiella pneumoniae* (CP-KP) has been challenging the global health system, associated with infections with high mortality rates [1,2]. Furthermore, CP-KP isolates are resistant to most of the antimicrobial agents available in clinical practice, restricting the therapeutic options [3,4]. Recently, new therapeutic options were made available, such as the new antibiotic cefiderocol (FDC), a siderophore cephalosporin [5], and novel β-lactam/β-lactamase inhibitor combinations (BLICs) such as ceftazidime–avibactam (CAZ-AVI), meropenem–vaborbactam (MVB), and imipenem–relebactam (IMR) [6]. In recent years, CAZ-AVI has become the most widely used antibiotic for the treatment of CP-KP infections [7]. However, with the increase in its clinical use and the consequent antibiotic selective pressure, KPC-producing *K. pneumoniae* (KPC-KP) isolates started to develop resistance to CAZ-AVI, mainly through mutation of the *bla*_KPC_ gene, leading to the inefficacy of the antibiotic [8]. Besides mutation of the *bla*_KPC_ gene, resistance to CAZ-AVI is due to several mechanisms, likely acting together, including the alterations of the major porins that allow the diffusion of CAZ-AVI across the outer membrane (OmpK35 and OmpK36 porins) and the increased gene expression and/or copy number of *bla*_KPC_ [9,10]. Isolates that produce KPC variants are usually susceptible to meropenem, which can be used as a last-resort antibiotic after the failure of CAZ-AVI treatment [11]. On the other hand, such isolates can exhibit cross-resistance to FDC, possibly due to the similar structure of the side chains between FDC and ceftazidime [12], with the contribution of other mechanisms including the alteration of porins. The mechanism underlying the resistance to FDC is a combination of different mechanisms including the co-expression of different β-lactamases (NDM metallo β-lactamases and KPC variants), mutations affecting siderophore–drug receptor expression/function (*cir*A and *fiu* of *Escherichia coli*), overexpression of efflux pumps (OmpK35 and OmpK36), and target modification (penicillin-binding protein 3, PBP-3 coded by the *fts*I gene) [13,14].

Here we describe the phenotypic and genomic analysis of a KPC-KP isolate harboring a novel KPC-3 variant, bearing an amino-acid insertion within the Ω-loop region. This KPC-KP isolate was resistant to CAZ-AVI and FDC and was obtained from a patient with urinary tract infection (UTI), previously exposed to CAZ-AVI treatment.

## 2. Results

### 2.1. Strain Collection

In October 2022, the national reference laboratory (NRL) for Antibiotic-Resistance (AMR) at Istituto Superiore di Sanità (ISS, Rome, Italy) received a CAZ-AVI-resistant KPC-KP obtained from a 31-year-old patient. In September 2022, the patient had been hospitalized for bloodstream infection (BSI) due to KPC-KP and ESBL-*E. coli* and UTI caused by KPC-KP, and he had received treatment with CAZ-AVI. After recovery, the patient was discharged with intestinal colonization by KPC-KP, susceptible to CAZ-AVI. Only the CAZ-AVI-resistant KPC-KP isolate (KP_RM_1) obtained from the UTI episode in October 2022 was available for further investigation. 

### 2.2. Antimicrobial Susceptibility Testing

Antibiotic susceptibility testing results for KP_RM_1 showed that the isolate was resistant to ertapenem but susceptible to meropenem and imipenem (Table 1). The isolate was resistant to all the β-lactams tested, quinolones, and aminoglycosides but it was susceptible to colistin, tigecycline, and trimethoprim/sulfamethoxazole. Concerning the BLICs, the isolate was resistant to CAZ-AVI and susceptible to MVB and IMR. Resistance to FDC (MIC ≥ 8 mg/L) was also detected. Isolates from the previous hospitalization in September for BSI and UTI were resistant to all the β-lactams tested and ciprofloxacin and susceptible to CAZ-AVI, except the UTI isolate, which was not tested (Table 1). *Klebsiella pneumoniae* isolates from BSI and UTI were also resistant to imipenem and meropenem. Resistance to FDC was not tested for those isolates (Table 1). 

### 2.3. Whole-Genome Sequencing and In Silico Analysis

KP_RM_1 was subjected to both Illumina and Oxford Nanopore Technologies (ONT) sequencing. WGS analysis integrating short and long reads produced a circular chromosome of 5,396,125 bp in size, with a GC content of 57.3%. Gene prediction and annotation identified 5019 coding sequences, 25 rRNAs, and 86 tRNAs. The isolate was assigned to ST101 by in silico multilocus sequence typing (MLST). The analysis of the capsular biosynthesis locus (KL) and O-locus revealed that the isolate was associated with the KL17 locus (*wzi*-137 allele) and the O1/O2v1 locus. The iron-scavenging siderophore yersiniabactin *ybt*9 gene cluster, associated with the mobile genetic element ICEKp3, was also detected, belonging to YbST183. Resistome analysis revealed the presence of several resistance determinants and gene mutations (Table 2). The isolate carried chromosomally encoded resistance gene *bla*_SHV-1_ along with quinolone-related resistance gene mutations, causing substitutions S83Y and D87N in GyrA and S80I in ParC, respectively. Regarding the major porins that allow the diffusion of CAZ-AVI across the outer membrane, the isolate had a truncated OmpK35 (at amino acid 63) and a mutated OmpK36 with a threonine and aspartic acid insertion at position 134 (134TD duplication). No tigecycline- or colistin-acquired resistance genes or chromosomal mutations associated with resistance to these antibiotics were found.

Siderophore receptor and *fts*I gene comparisons with the reference genome ATCC13883 revealed that *cir*A possessed a mutation, leading to an amino acid substitution in CirA protein at position 134 (A134V); the *fiu* gene had a mutation that resulted in an amino acid substitution in the Fiu protein at position 387 (D387N); the *fts*I gene was 100% identical to the reference gene.

### 2.4. Plasmid Analysis

**Figure 1:**
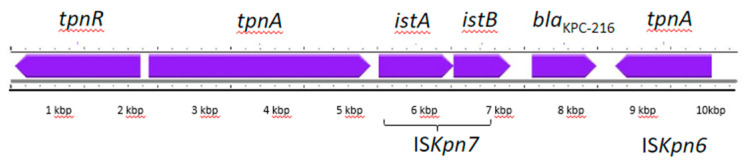
Schematic representation of Tn*4401* structure identified on plasmid P007 from *K. pneumoniae* isolate KP_RM_1. Genes are represented as purple arrows with their corresponding transcription orientation. Bar scale indicates the length in kbp.

Plasmid analysis revealed the presence of five plasmids (Table 3): four small Col-type plasmids (range 5–9 kb in size) and a larger plasmid (P007). Only the largest plasmid harbored resistance genes. Plasmid P007, with a length of 72,398 bp containing 80 CDS, carried a mutated *bla*_KPC-3_. This *bla*_KPC_ gene variant had a triplet AAG insertion at position 504 with respect to the *bla*_KPC-3_ gene, corresponding to the insertion of the amino acid lysin (K) at position 168 (+K168), generating a new KPC-3 variant named KPC-216 (Supplementary Figure S1). This insertion occurred in the active site of KPC within the Ω-loop (contained within amino acids Arg164 and Asp179). The *bla*_KPC-216_ gene was located on Tn*4401*, a Tn*3*-like transposon (approximately 10 kb), which contains *tnpA* transposase, a *tnpR* resolvase, and insertion sequences (IS) upstream and downstream from the *bla*_KPC_ gene (IS*Kpn7* and IS*Kpn6*, respectively) (Figure 1).

In addition, P007 had other antibiotic resistance determinants: gene coding for resistance to aminoglycosides (*armA*) and macrolides, lincosamides, and streptogramins (*msr*(E) and *mph*(E) (Table 3)). The *mer* operon, conferring resistance to mercuric ions, was also present in P007.

P007 was a multi-replicon plasmid, carrying IncFIA(HI1), IncFII(K) (belonging to pMLST [K2:A13:B-]), and IncR.

Comparative analysis of the P007 plasmid performed using BLASTN and showing the best match revealed that it had 99.9% identity and 100% coverage with two plasmids (GenBank accession number MT809698.1 and MT809692.1) from *K. pneumoniae* strains isolated in Italy [15]. 

Conjugation experiments with the KP_RM_1 isolate carried out to assess the transfer potential of P007 failed to produce transconjugants when rifampicin-resistant *E. coli* DH5α was used as the recipient strain. 

### 2.5. Virulence Factors Analysis

Several genes coding for virulence factors (VFs) were detected in the KP_RM_1 isolate, as shown in Table 4. Genes coding for VFs involved in adhesion (Types I, III, and IV fimbriae), antiphagocytosis (capsule), efflux pump (*acr*AB), iron uptake (aerobactin, ent siderophore, Salmochelin, and Yersiniabactin), the secretion system (T6SS-I/-II/-III), serum resistance (LPS), and fimbrial adherence (Stb) were detected. Virulence genes coding for VFs related to toxicity were not found in this isolate.

## 3. Discussion

The introduction of the BLICs into clinical practice represented a new and effective therapeutic alternative to combat serious carbapenemase-resistant Enterobacterales infections [16,17]. However, resistance was soon reported, mainly due to mutations of the *bla*_KPC-2_ or *bla*_KPC-3_ genes, bringing new challenges to clinical treatment [18]. Recently, a plethora of new KPC variants were described, originating from amino acid substitution, insertion, or deletion in the active sites of wild-type *bla*_KPC_ [19]. These variants produce carbapenemases showing a modified three-dimensional structure that has reduced activity on CAZ-AVI, associated with resistance to one of the last antimicrobial agents available for the treatment of carbapenem-resistant Enterobacterales infections. In the present study, we analyzed a KPC-*K. pneumoniae* strain (KP_RM_1) resistant to CAZ-AVI carrying a novel *bla*_KPC_ that was isolated from a critically ill patient presenting with UTI. The KPC variant, named KPC-216, is a single amino acid variant of KPC-3, carrying a lysin insertion at position 168 (+K168), along with mutations of porin OmpK36 and the deletion of OmpK35. The modifications of porins possibly restrict antibiotic entry and act in synergy with variant carbapenemase enzymes to increase the level of resistance to CAZ-AVI [20]. The insertion occurred in the KPC active site, within the Ω-loop, frequently associated with mutations leading to resistance to CAZ-AVI [21]. As reported in other studies, we observed cross-resistance between CAZ-AVI and FDC and the restoration of susceptibility to imipenem and meropenem [22,23]. This latter side-effect is an important observation for clinicians who can use meropenem in association with other antimicrobial agents as a salvage therapy after failure of CZA treatment.

In this strain, FDC resistance may be due to a combination of factors, such as the presence of a KPC variant, the loss of OmpK35, and mutations in OmpK36 and the siderophores CirA and Fiu.

The *bla*_KPC-216_ gene was carried on an IncF plasmid, as frequently reported in most KPC-carrying plasmids, and it was embedded into a Tn*4401* transposon [24,25]. The plasmid P007 harboring the blaKPC-216 gene was characterized by the presence of both FIA(HI1) and R replicon in an ST101 strain, as previously reported in a paper describing new variants *bla*_KPC-39_ and *bla*_KPC-68_, conferring resistance to CAZ-AVI [15]. Strains carrying *bla*_KPC-39_ and *bla*_KPC-68_ were isolated in a different hospital in the same city in 2019 three years before the isolation of KP_RM_1 [15]. *bla*_KPC-39_- and *bla*_KPC-68_-associated plasmids were almost identical to P007 and lacked the entire transfer locus *tra*, essential for transfer during conjugation. Likely, for that reason, conjugation experiments of the P007 plasmid were unsuccessful, as previously reported [26]. Also in our study, the *bla*_KPC_ gene was found in an isolate belonging to ST101, a high-risk clone causing hospital infections and outbreaks that is, nowadays, one of the predominant high-risk lineages circulating in Italy [27]. 

The patient was treated with CAZ-AVI for a BSI during a previous hospitalization and had intestinal colonization by KPC-KP before the isolation of KP_RM_1 resistant to CAZ-AVI. Unfortunately, the KPC-KP isolates causing BSI and UTI had not been stored and therefore it is impossible to know if the KPC variant was already present, but they were resistant to meropenem and susceptible to CAZ-AVI. It is likely that the prolonged use of CAZ-AVI in the previous month might have selected the emergence of the CAZ-AVI-resistant isolate.

The presence of a plasmid-borne KPC-variant in a high-risk clone is alarming in a country such as Italy with one of the highest levels of CR-KP circulation, where further spreading of plasmids and clones by horizontal transfer of plasmids or mobility of small genetic elements as transposons could worsen the scenario. Critically ill patients who are frequently subjected to repeated treatments with different antibiotics for long periods are particularly prone to the selection of AMR bacterial pathogens that can acquire new resistance mechanisms. Moreover, *K. pneumoniae* can colonize fragile patients for a long period, as already shown in previous studies [28,29], and can accumulate resistance genes under selective pressure. 

This study presents some limitations: (i) the previous isolates from BSI and UTI episodes in the preceding month from the same patient were not available for genomic comparison to evaluate in-host changes in *K. pneumoniae* isolates over time. This still leaves open the possibility that the KPC variant was not necessarily selected by the previous use of CAZ-AVI, resulting in CAZ-AVI resistance within the same isolate. Therefore, the KPC variant could potentially arise from the acquisition of a new isolate with a different susceptibility pattern, likely during the previous hospitalization, though this hypothesis is less feasible than the previous one. Furthermore, (ii) our attempt to transfer the plasmid to a recipient strain was not successful, possibly due to the lack of the *tra* locus in P007. 

## 4. Materials and Methods

### 4.1. Phenotypic Analysis

In October 2022, the NRL for AMR at Istituto Superiore di Sanità received a KPC-KP isolate resistant to CAZ-AVI obtained from the UTI of a 31-year-old patient. The patient had been admitted to different hospitals several times in the previous 6 months for his chronic conditions (chronic renal failure, chronic obstructive pulmonary disease, hepatitis C virus positivity, and latent tuberculosis). In September 2022, the patient had been hospitalized for BSI due to KPC-KP and ESBL-*E. coli* and UTI caused by KPC-KP. He then received treatment with CAZ-AVI (2/0.5 g intravenous every 8 h for 11 days). Those isolates had not been stored but bacterial identification and in vitro susceptibility testing using the VITEK-MS 2 system (bioMérieux, Craponne, France) were performed at the San Filippo Neri hospital laboratory located in Rome. The KPC-KP isolate, designated as KP_RM_1, was then sent to the NRL for AMR at ISS for further phenotypic and molecular characterization. Carbapenemase production was confirmed by phenotypic testing with the agar tablet/disk diffusion method (KPC/MBL and OXA-48 Confirm Kit; ROSCO Diagnostica, Taastrup, Denmark). Antibiotic susceptibility testing was confirmed by the reference broth microdilution method using lyophilized, custom microtitration plates (Thermo Fisher Scientific, Rodano, Italy), including MVB, IMR, and FDC. *E. coli* ATCC 25922 and *Klebsiella pneumoniae* ATCC BAA-2814 were used as the quality control strains. The interpretative breakpoints were based on EUCAST, version 14.0 [30]. For tigecycline, the results were interpreted using the *E. coli* breakpoints.

To assess the transfer potential of P007, conjugation experiments were performed using KP_RM_1 as the donor and rifampicin-resistant *E. coli* DH5-α as the recipient. Transconjugants were selected on MH agar plates, supplemented with rifampicin (50 mg/L) and ceftazidime (4 mg/L) at 37 °C.

### 4.2. Whole-Genome Sequencing and In Silico Analysis

The KPC-KP isolate was submitted to whole-genome sequencing: genomic DNA was extracted from an overnight culture grown in Muller Hinton Broth by using the NucleoSpin^®^DNA Extraction Kit (Macherey-Nagel, Duren, Germany) according to the manufacturer’s instructions. Both short-read and long-read sequencing was performed. A genomic DNA paired-end library was produced using the Nextera XT DNA sample preparation kit (Illumina Inc., San Diego, CA, USA) and was sequenced on the Illumina NextSeq system (Illumina Inc.) to obtain short-reads. The library used to obtain long reads was prepared with the Rapid Barcoding kit 96 and sequenced using the MinION Mk1C sequencing platform (Oxford Nanopore Technologies, Oxford, UK). De novo assembly of short reads was performed using SPAdes 3.14 software through the ARIES public Galaxy server (https://w3.iss.it/site/aries/, accessed on 18 February 2024). We integrated Illumina assemblies and Oxford Nanopore Technologies reads by using the Unicycler hybrid assembly tool [31]. We ensured that the genome was free of contamination by using KmerFinder, a tool available at the Center for Genomic Epidemiology (CGE) server (https://cge.cbs.dtu.dk/services/, accessed on 2 April 2024). We confirmed the identification of the isolate as *K. pneumoniae sub. pneumoniae* by using FastANI (Galaxy version 1.3) through the European Galaxy server (https://usegalaxy.eu/, accessed on 10 May 2024). In silico analysis of the assembled contigs was also performed using tools available at CGE and screened for plasmid content using the PlasmidFinder tool. Plasmids of the IncF type were subtyped by assigning a replicon allele using the pMLST tool at CGE. Gene prediction and annotation of assembled chromosomes and plasmids were performed by using PROKKA (Galaxy Version 1.14.5), through the European Galaxy server. Proksee was used to visualize and draw the transposon associated with the *bla*_KPC_ gene (https://proksee.ca/, assessed on 10 May 2024). The identification of resistance and capsular virulence genes related to KPC-KP was performed by Kleborate using the ARIES Galaxy server (Galaxy Version 2.3.0). In silico analysis also permitted us to determine if each AMR gene was located on the chromosome or on a plasmid by searching for AMR genes in each sequence separately (i.e., chromosome or each plasmid). We then submitted the nucleotide sequence of the *bla*_KPC_ gene to GenBank to obtain the allele curation for the designation of the new KPC-variant (https://www.ncbi.nlm.nih.gov/pathogens/submit-beta-lactamase/, accessed on 18 February 2024). 

Genes *cir*A, *fiu*, and *fts*I were compared by BLAST at the National Center for Biotechnology Information (NCBI) (https://blast.ncbi.nlm.nih.gov/Blast.cgi, accessed on 18 February 2024) with the *K. pneumoniae* ATCC 13883 reference genome (GenBank accession no. JOOW00000000) to identify mutations resulting in the alteration of siderophore receptors or PBP-3, possibly associated with FDC resistance. Comparative analysis of the P007 plasmid was performed using BLASTN (Megablast) at NCBI. The identification of virulence genes related to KP_RM_1 was performed by searching the virulence factor database (VFDB) (http://www.mgc.ac.cn/VFs/main.htm, accessed on 7 May 2024).

## 5. Conclusions

In conclusion, we report the identification of *bla*_KPC-216_, a new plasmid-borne *bla*_KPC-3_ variant associated with CAZ-AVI resistance and cross-resistance to FDC. Considering the broad use of CAZ-AVI, when dealing with infection caused by KPC-KP, susceptibility to CAZ-AVI and FDC should be tested before their clinical use. The KPC variants associated with CAZ-AVI resistance should be determined to promptly inform clinicians and start the appropriate antimicrobial therapy. From this perspective and due to the complexity of interactions between co-produced efflux pump variants and variant carbapenemases, WGS should support routine diagnostics to investigate the resistance mechanisms of AMR pathogens more deeply.

## Figures and Tables

**Table 1:** Antimicrobial resistance profile of *Klebsiella pneumoniae* and *Escherichia coli* isolates from the patient.

Antibiotics	MIC (mg/L)/Susceptibility Category ^1^							
	KP_RM_1 (October 2022)		KP_RM_BSI (September 2022)		KP_RM_UTI (September 2022)		EC ^2^_RM_BSI (September 2022)	
Amikacin	≥32	R	na ^3^	na	na	na	na	na
Ampicillin	≥16	R	na	na	na	na	na	na
Amoxicillin/clavulanic acid	16/2	R	≥32	R	≥32	R	≥32	R
Cefepime	≥8	R	≥32	R	≥32	R	16	R
Ceftazidime	≥8	R	≥64	R	≥64	R	≥64	R
Ceftazidime/avibactam (CAZ-AVI)	16/4	R	4	S	na	na	≤0.12	S
Cefotaxime	≥4	R	na	na	na	na	na	na
Cefiderocol (FDC)	≥8	R	na	na	na	na	na	na
Ciprofloxacin	≥1	R	≥4	R	≥4	R	≥4	R
Colistin	≤0.5	S	0.5	S	na	na	0.5	S
Ertapenem	≥2	R	na	na	na	na	na	na
Imipenem	≤1	S	≥16	R	8	R	≤0.125	S
Imipenem–relebactam (IMR)	0.25/4	S	na	na	na	na	na	na
Gentamicin	≥8	R	≥16	R	≥16	R	≥16	R
Levofloxacin	≥2	R	na	na	na	na	na	na
Meropenem	2	S	≥16	R	≥16	R	≤0.125	S
Meropenem–vaborbactam (MVB)	0.5/8	S	4	S	na	na	na	na
Nitrofurantoin	≥64	R	na	na	na	na	na	na
Piperacillin/tazobactam	16/4	R	≥128	R	≥128	R	8	S
Tigecycline	≤0.5	S	na	na	na	na	na	na
Tobramycin	≥8	R	≥16	R	na	na	≥16	R
Trimethoprim/sulphamethoxazole	≤1/19	S	≤20	S	≤20	S	≥320	R

^1^ R, resistant; S, susceptible; ^2^ EC, *E. coli*; ^3^ na, not available.

**Table 2:** Resistome analysis of *Klebsiella pneumoniae* KP_RM_1.

	Resistant to	Localization
Resistance gene		
*armA*	Aminoglycosides	Plasmid
*bla*_SHV-1_	β-lactams	Chromosome
*bla*_KPC-216_	β-lactams	Plasmid
*msr*(E)	Macrolides, lincosamides and streptogramins (MLS)	Plasmid
*mph*(E)	Macrolides, lincosamides and streptogramins (MLS)	Plasmid
Protein substitution		
GyrA S83Y	Quinolones	Chromosome
GyrA D87N	Quinolones	Chromosome
ParC S80I	Quinolones	Chromosome
OmpK35 AA63 stop	β-lactams	Chromosome
OmpK36 134TD duplication	β-lactams	Chromosome

**Table 3:** Plasmid analysis of *Klebsiella pneumoniae* KP_RM_1.

Plasmid ID	Length (bp)	Plasmid Replicon	pMLST	Resistance Genes
P003	5356	Col440II		-
P014	7585	Col156		-
P026	9289	ColRNAI		-
P040	5592	ColRNAI		-
P007	72,398	IncFIA(HI1), IncFII(K), IncR	[K2:A13:B-]	*bla*_KPC-216_, *armA*, *mph*(E), *msr*(E)

**Table 4:** Virulence factors and related genes by VF class in isolate KP_RM_1.

VFclass	Virulence Factors	Related Genes
Adherence	Type 3 fimbriae	*mrkA*, *mrkB*, *mrkC*, *mrkD*, *mrkF*, *mrkH*, *mrkI*, *mrkJ*
	Type I fimbriae	*fimA*, *fimB*, *fimC*, *fimD*, *fimE*, *fimF*, *fimG*, *fimH*, *fimI*, *fimK*
	Type IV pili (Yersinia)	*pilW*
Antiphagocytosis	Capsule	*wiz*
Efflux pump	AcrAB	*acrA*, *acrB*
Iron uptake	Aerobactin	*iutA*
	Ent siderophore	*entA*, *entB*, *entC*, *entD*, *entE*, *entF*, *entS*, *fepA*, *fepB*, *fepC*, *fepD*, *fepG*, *fes*
	Salmochelin	*iroE*, *iroN*
	Yersiniabactin	*fyuA*, *irp1*, *irp2*, *ybtA*, *ybtE*, *ybtP*, *ybtQ*, *ybtS*, *ybtT*, *ybtU*, *ybtX*
Regulation	RcsAB	*rcsA*, *rcsB*
Secretion system	T6SS-I	*clpV/tssH*, *dotU/tssL*, *hcp/tssD*, *icmF/tssM*, *impA/tssA*, *ompA*, *sciN/tssJ*, *tssF*, *tssG*, *vasE/tssK*, *vgrG/tssI*, *vipA/tssB*, *vipB/tssC*
	T6SS-II	*clpV*, *impH*, *impJ*, *sciN*, *vasA/impG*
	T6SS-III	*dotU*, *icmF*, *impA*, *impF*, *impG*, *impH*, *impJ*, *ompA*, *sciN*, *vgrG*
Serum resistance	LPS rfb locus	*rfb*
Fimbrial adherence determinants	Stb (Salmonella)	*stbA*, *stbB*, *stbC*, *stbD*

## Data Availability

Sequence data were submitted to GenBank at NCBI under the BioProject PRJNA1029903; KPC-216 Accession No. PP379169.

## Supplementary Materials

The following supporting information can be downloaded at: https://www.mdpi.com/article/10.3390/antibiotics13060507/s1, Figure S1: (A) Nucleotide alignment of blaKPC-216 in comparison to blaKPC-3 gene; (B) Aminoacid alignment of KPC-216 in comparison to KPC-3 protein.
