# Supplementary material for: Detection of KPC-216, a Novel KPC-3 Variant, in a Clinical Isolate of Klebsiella pneumoniae ST101 Co-Resistant to Ceftazidime-Avibactam and Cefiderocol
Source: Antibiotics (Basel). 2024 May 29;13(6):507. doi: 10.3390/antibiotics13060507 (PMC11200853; doi:10.3390/antibiotics13060507)
Supplement: Supplementary file 1 [file antibiotics-13-00507-s001.zip › antibiotics-3002293-supplementary.pdf]

A. Nucleotide alignment

```
blaKPC-216  1    ATGTCACTGTATCGCCGTCTAGTTCTGCTGTCTTGTCTCTCATGGCCGCTGGCTGGCTTT  60
blaKPC-3    1    ............................................................  60

blaKPC-216  61   TCTGCCACCGCGCTGACCAACCTCGTCGCGGAACCATTCGCTAAACTCGAACAGGACTTT  120
blaKPC-3    61   ............................................................  120

blaKPC-216  121  GGCGGCTCCATCGGTGTGTACGCGATGGATACCGGCTCAGGCGCAACTGTAAGTTACCGC  180
blaKPC-3    121  ............................................................  180

blaKPC-216  181  GCTGAGGAGCGCTTCCCACTGTGCAGCTCATTCAAGGGCTTTCTTGCTGCCGCTGTGCTG  240
blaKPC-3    181  ............................................................  240

blaKPC-216  241  GCTCGCAGCCAGCAGCAGGCCGGCTTGCTGGACACACCCATCCGTTACGGCAAAAATGCG  300
blaKPC-3    241  ............................................................  300

blaKPC-216  301  CTGGTTCCGTGGTCACCCATCTCGGAAAAATATCTGACAACAGGCATGACGGTGGCGGAG  360
blaKPC-3    301  ............................................................  360

blaKPC-216  361  CTGTCCGCGGCCGCCGTGCAATACAGTGATAACGCCGCCGCCAATTTGTTGCTGAAGGAG  420
blaKPC-3    361  ............................................................  420

blaKPC-216  421  TTGGGCGGCCCGGCCGGGCTGACGGCCTTCATGCGCTCTATCGGCGATACCACGTTCCGT  480
blaKPC-3    421  ............................................................  480

blaKPC-216  481  CTGGACCGCTGGGAGCTGGAGCTGAAGAACTCCGCCATCCCAGGCGATGCGCGCGATACC  540
blaKPC-3    481  .......................---..................................  537

blaKPC-216  541  TCATCGCCGCGCGCCGTGACGGAAAGCTTACAAAAACTGACACTGGGCTCTGCACTGGCT  600
blaKPC-3    538  ............................................................  597

blaKPC-216  601  GCGCCGCAGCGGCAGCAGTTTGTTGATTGGCTAAAGGGAAACACGACCGGCAACCACCGC  660
blaKPC-3    598  ............................................................  657

blaKPC-216  661  ATCCGCGCGGCGGTGCCGGCAGACTGGGCAGTCGGAGACAAAACCGGAACCTGCGGAGTG  720
blaKPC-3    658  ............................................................  717

blaKPC-216  721  TATGGCACGGCAAATGACTATGCCGTCGTCTGGCCCACTGGGCGCGCACCTATTGTGTTG  780
blaKPC-3    718  ............................................................  777

blaKPC-216  781  GCCGTCTACACCCGGGCGCCTAACAAGGATGACAAGTACAGCGAGGCCGTCATCGCCGCT  840
blaKPC-3    778  ............................................................  837

blaKPC-216  841  GCGGCTAGACTCGCGCTCGAGGGATTGGGCGTCAACGGGCAGTAA  885
blaKPC-3    838  .............................................  882
```

B. Aminoacid alignment

```
KPC-216  1    MSLYRRLVLLSCLSWPLAGFSATALTNLVAEPFAKLEQDFGGSIGVYAMDTGSGATVSYR  60
KPC-3    1    ............................................................  60

KPC-216  61   AEERFPLCSSFKGFLAAAVLARSQQQAGLLDTPIRYGKNALVPWSPISEKYLTTGMTVAE  120
KPC-3    61   ............................................................  120

KPC-216  121  LSAAAVQYSDNAAANLLLKELGGPAGLTAFMRSIGDTTFRLDRWELELKNSAIPGDARDT  180
KPC-3    121  ................................................-...........  179

KPC-216  181  SSPRAVTESLQKLTLGSALAAPQRQQFVDWLKGNTTGNHRIRAAVPADWAVGDKTGTCGV  240
KPC-3    180  ............................................................  239

KPC-216  241  YGTANDYAVVWPTGRAPIVLAVYTRAPNKDDKYSEAVIAAAARLALEGLGVNGQ  294
```

```
KPC-3    240  .....................................................  293
```

Supplementary Figure S1. A. Nucleotide alignment of *bla*$_{KPC-216}$ in comparison to *bla*$_{KPC-3}$ gene; B. Aminoacid alignment of KPC-216 in comparison to KPC-3 protein. The insertion of a triplet/aminoacid is highlighted in grey. The Ω-loop is underlined in aminoacid alignment.
